# Editorial: Zebrafish as a translational neuroscience model: today’s science and tomorrow’s success

**DOI:** 10.3389/fphys.2023.1202198

**Published:** 2023-04-19

**Authors:** Wael M. Y. Mohamed, Marc Ekker

**Affiliations:** ^1^ Basic Medical Science Department, Kulliyyah of Medicine, International Islamic University Malaysia, Kuantan, Pahang, Malaysia; ^2^ Department of Biology, University of Ottawa, Ottawa, ON, Canada

**Keywords:** zebrafish, translational, neuroscience, model, science

## Zebrafish as a promising neuroscience model

One of the most significant developments in modern science is the rise of translational medicine. *Danio rerio* has offered a new venue for important studies, especially in the field of neurodevelopmental research. Benefits have been shown in the treatment of many neurodegenerative disorders, including Parkinson’s disease, Alzheimer’s disease, and Huntington’s disease. Zebrafish have made molecular investigations, especially the omics, feasible in a manner that was previously impossible using other animal models, such as primates or rats, because of their translucent embryos, amenability to genetic modifications, and short generation time. Nowadays, zebrafish serve as a great model to analyse neurobehavioral traits that are relevant to humans. The fields of biology, neurology, pharmacology, and toxicology all make use of it.

The world of model organisms has increasingly acquired interest in this small tropical vertebrate species. Zebrafish have further general strengths: economic efficiency, high fertility. These characteristics have given researchers an unparalleled vertebrate experimental system with a capacity for live biological imaging and genetic and drug screening. Zebrafish were used in neuroscience and developmental genetics because of their high levels of genetic, anatomical, and physiological resemblance with humans. They have also been used to study human diseases. In 2013, the publication of the Zebrafish reference genome accelerated disease modeling in this organism. More than 80% of the disease-causing human proteins have an ortholog in zebrafish. Furthermore, new therapeutic targets and molecules were identified or discovered using zebrafish that are now being considered for human trials or are waiting for clinical application. Nevertheless, more zebrafish models are needed to broaden our understanding of human diseases. This Research Topic offers updated insights from researchers from around the world on zebrafish as a model of translational neuroscience research. While the zebrafish brain and the human brain are noticeably different in size and shape from one another, they share a very comparable organisational structure. Several regions of the zebrafish brain seem to be connected to and are often remarkably conserved when compared to their human counterparts. The ventral telencephalon of zebrafish, for instance, is thought to be similar to the human striatum. Moreover, zebrafish traits and behaviours may serve as useful models for human behaviour and development. The movement deficits seen in zebrafish exposed to neurotoxins are highly predictive of the bradykinesia seen by people with Parkinson’s disease (Hughes et al., 2020).

Genetic analyses have shown that there is 70% sequence similarity between the zebrafish and human genomes (Shehwana and Konu, 2019), with 80% of the genes positioned on the same chromosomes and in the same orders (Howe et al., 2013). Transgenic models in zebrafish are well-documented, and their genetics have been extensively studied (Kalueff and Cachat, 2011). Furthermore, the zebrafish is a unique model since its embryos grow externally and are transparent, allowing researchers to see their progress in real time as they occur (Rahman Khan and Sulaiman Alhewairini, 2019).

In this Research Topic, Pakdaman et al. examined the effects of impaired CHIP ubiquitin ligase activity in zebrafish (*D. rerio*). They characterized the zebrafish *stub1* gene and Chip protein, generated and characterized a zebrafish mutant causing truncation of the Chip functional U-box domain. Mutant fish had decreased total 26S proteasome activity in the brain and showed behavioral changes. They concluded that truncation of the Chip U-box domain leads to impaired ubiquitin ligase activity, resulting inbehavioral and anatomical changes in zebrafish. This illustrates the potential of zebrafish to study *STUB1*-mediated diseases. Similarly, Heylen et al. tried to understand the mechanistic processes underlying the changes of brain tissue and networks toward increased seizure susceptibility using zebrafish larvae. They administred kainic acid (KA) by pericardial injection at an early zebrafish larval stage. Then the epileptic phenotype induced was examined by quantification of seizure-like behavior using automated video recording. Epileptiform brain activity was measured via local field potential (LFP) recordings. They found that KA induced a massive damage and inflammation in the zebrafish brain and seizure-like locomotor behavior. They described a chemically-induced larval zebrafish epilepsy model offering unique insights into studying epileptogenic processes *in vivo* and suitable for high-throughput Anti-epileptic drugs (AEDs) screening purposes and rapid genetic investigations. Further, Martin et al. worked on the larval ZF and they used Single-Cell RNA Sequencing to characterize the Molecular Heterogeneity of the Larval Zebrafish Optic Tectum (OT). They concluded that the larval zebrafish OT is a complex structure containing at least 25 transcriptionally distinct cell populations. This is the first time scRNA-seq has been applied to explore the OT alone and in depth. Finally, this current SI includes a narrative review by Singh and Patten that is highlighted the modeling neuromuscular diseases in zebrafish. They described the generation of different zebrafish genetic models mimicking NMDs and how they are used for drug discovery and therapy development. They recommended that optogenetic approaches to modulate subcellular expression and structure of molecules *in vivo* in zebrafish larvae can lead to the discovery of novel pathways, mechanistic links and new therapeutic interventions. New zebrafish models are continuously developed to study the development, progression and severity of common and rare NMDs, which can be further used for targeted gene therapy and drug discovery.

In the current Research Topic, we highlighted the use of zebrafish as a model for the evaluation of potential new treatments for neurological illnesses. Key morphological, physiological, and biochemical abnormalities in certain classes of neurons are triggered by the gene knockdown of orthologous zebrafish or transgenic expression of pathogenic genes related with human neurodegenerative illnesses. This points to a high degree of functional conservation between human genes associated with neurodegenerative diseases and their zebrafish orthologs. Hence, zebrafish may serve as a useful alternative model for understanding the molecular basis of PD. Considering the zebrafish’s unique traits, we anticipate a rise in its popularity as a high-throughput drug-screening vertebrate platform. In line with the idea of precision medicine, having a thorough understanding of the omics (genomics, proteomics, and metabolomics) of an illness might aid healthcare professionals in personalising treatment plans. To this end, zebrafish have been and will continue to be one of the gold standards of neuroscience models, particularly for projects requiring molecular research.
